# Supplementary material for: HIVprotI: an integrated web based platform for prediction and design of HIV proteins inhibitors
Source: J Cheminform. 2018 Mar 9;10:12. doi: 10.1186/s13321-018-0266-y (PMC5845081; doi:10.1186/s13321-018-0266-y)
Supplement: Supplementary file 2 — Additional file 2. Source code of HIVProtI web server. [file 13321_2018_266_MOESM2_ESM.zip › HIVprotI_Source-code/hivprotI/weka-3-6-6/doc/weka/gui/explorer/AttributeSelectionPanel.html]

AttributeSelectionPanel


---


|  |  |  |
| --- | --- | --- |
| **Overview** \| **Package** \| **Class** \| **Tree** \| **Deprecated** \| **Index** \| **Help** \| **Weka's home** | |  |
| **PREV CLASS**   **NEXT CLASS** | **FRAMES**    **NO FRAMES**     **All Classes** |  |
| SUMMARY: NESTED \| FIELD \| CONSTR \| METHOD | DETAIL: FIELD \| CONSTR \| METHOD |  |


---


## weka.gui.explorer Class AttributeSelectionPanel

```
java.lang.Object
  java.awt.Component
      java.awt.Container
          javax.swing.JComponent
              javax.swing.JPanel
                  weka.gui.explorer.AttributeSelectionPanel
```

**All Implemented Interfaces:**: java.awt.image.ImageObserver, java.awt.MenuContainer, java.io.Serializable, java.util.EventListener, javax.accessibility.Accessible, Explorer.CapabilitiesFilterChangeListener, Explorer.ExplorerPanel, Explorer.LogHandler

---

``` public class AttributeSelectionPanel extends javax.swing.JPanel implements Explorer.CapabilitiesFilterChangeListener, Explorer.ExplorerPanel, Explorer.LogHandler ```

This panel allows the user to select and configure an attribute
evaluator and a search method, set the
attribute of the current dataset to be used as the class, and perform
attribute selection using one of two selection modes (select using all the
training data or perform a n-fold cross validation---on each trial
selecting features using n-1 folds of the data).
The results of attribute selection runs are stored in a results history
so that previous results are accessible.

**Version:**
:   $Revision: 7101 $

**Author:**
:   Mark Hall (mhall@cs.waikato.ac.nz)

**See Also:**: Serialized Form

---

**Nested Class Summary**

**Nested classes/interfaces inherited from class javax.swing.JComponent**

`javax.swing.JComponent.AccessibleJComponent`

**Nested classes/interfaces inherited from class java.awt.Component**

`java.awt.Component.BaselineResizeBehavior`


**Field Summary**

**Fields inherited from class javax.swing.JComponent**

`TOOL_TIP_TEXT_KEY, UNDEFINED_CONDITION, WHEN_ANCESTOR_OF_FOCUSED_COMPONENT, WHEN_FOCUSED, WHEN_IN_FOCUSED_WINDOW`

**Fields inherited from class java.awt.Component**

`BOTTOM_ALIGNMENT, CENTER_ALIGNMENT, LEFT_ALIGNMENT, RIGHT_ALIGNMENT, TOP_ALIGNMENT`

**Fields inherited from interface java.awt.image.ImageObserver**

`ABORT, ALLBITS, ERROR, FRAMEBITS, HEIGHT, PROPERTIES, SOMEBITS, WIDTH`


**Constructor Summary**

`AttributeSelectionPanel()`
  
          Creates the classifier panel


| **Method Summary** | |
| --- | --- |
| `void` | `capabilitiesFilterChanged(Explorer.CapabilitiesFilterChangeEvent e)`             method gets called in case of a change event |
| `Explorer` | `getExplorer()`             returns the parent Explorer frame |
| `java.lang.String` | `getTabTitle()`             Returns the title for the tab in the Explorer |
| `java.lang.String` | `getTabTitleToolTip()`             Returns the tooltip for the tab in the Explorer |
| `static void` | `main(java.lang.String[] args)`             Tests out the attribute selection panel from the command line. |
| `void` | `setExplorer(Explorer parent)`             Sets the Explorer to use as parent frame (used for sending notifications about changes in the data) |
| `void` | `setInstances(Instances inst)`             Tells the panel to use a new set of instances. |
| `void` | `setLog(Logger newLog)`             Sets the Logger to receive informational messages |

**Methods inherited from class javax.swing.JPanel**

`getAccessibleContext, getUI, getUIClassID, setUI, updateUI`

**Methods inherited from class javax.swing.JComponent**

`addAncestorListener, addNotify, addVetoableChangeListener, computeVisibleRect, contains, createToolTip, disable, enable, firePropertyChange, firePropertyChange, firePropertyChange, getActionForKeyStroke, getActionMap, getAlignmentX, getAlignmentY, getAncestorListeners, getAutoscrolls, getBaseline, getBaselineResizeBehavior, getBorder, getBounds, getClientProperty, getComponentPopupMenu, getConditionForKeyStroke, getDebugGraphicsOptions, getDefaultLocale, getFontMetrics, getGraphics, getHeight, getInheritsPopupMenu, getInputMap, getInputMap, getInputVerifier, getInsets, getInsets, getListeners, getLocation, getMaximumSize, getMinimumSize, getNextFocusableComponent, getPopupLocation, getPreferredSize, getRegisteredKeyStrokes, getRootPane, getSize, getToolTipLocation, getToolTipText, getToolTipText, getTopLevelAncestor, getTransferHandler, getVerifyInputWhenFocusTarget, getVetoableChangeListeners, getVisibleRect, getWidth, getX, getY, grabFocus, isDoubleBuffered, isLightweightComponent, isManagingFocus, isOpaque, isOptimizedDrawingEnabled, isPaintingForPrint, isPaintingTile, isRequestFocusEnabled, isValidateRoot, paint, paintImmediately, paintImmediately, print, printAll, putClientProperty, registerKeyboardAction, registerKeyboardAction, removeAncestorListener, removeNotify, removeVetoableChangeListener, repaint, repaint, requestDefaultFocus, requestFocus, requestFocus, requestFocusInWindow, resetKeyboardActions, reshape, revalidate, scrollRectToVisible, setActionMap, setAlignmentX, setAlignmentY, setAutoscrolls, setBackground, setBorder, setComponentPopupMenu, setDebugGraphicsOptions, setDefaultLocale, setDoubleBuffered, setEnabled, setFocusTraversalKeys, setFont, setForeground, setInheritsPopupMenu, setInputMap, setInputVerifier, setMaximumSize, setMinimumSize, setNextFocusableComponent, setOpaque, setPreferredSize, setRequestFocusEnabled, setToolTipText, setTransferHandler, setVerifyInputWhenFocusTarget, setVisible, unregisterKeyboardAction, update`

**Methods inherited from class java.awt.Container**

`add, add, add, add, add, addContainerListener, addPropertyChangeListener, addPropertyChangeListener, applyComponentOrientation, areFocusTraversalKeysSet, countComponents, deliverEvent, doLayout, findComponentAt, findComponentAt, getComponent, getComponentAt, getComponentAt, getComponentCount, getComponents, getComponentZOrder, getContainerListeners, getFocusTraversalKeys, getFocusTraversalPolicy, getLayout, getMousePosition, insets, invalidate, isAncestorOf, isFocusCycleRoot, isFocusCycleRoot, isFocusTraversalPolicyProvider, isFocusTraversalPolicySet, layout, list, list, locate, minimumSize, paintComponents, preferredSize, printComponents, remove, remove, removeAll, removeContainerListener, setComponentZOrder, setFocusCycleRoot, setFocusTraversalPolicy, setFocusTraversalPolicyProvider, setLayout, transferFocusBackward, transferFocusDownCycle, validate`

**Methods inherited from class java.awt.Component**

`action, add, addComponentListener, addFocusListener, addHierarchyBoundsListener, addHierarchyListener, addInputMethodListener, addKeyListener, addMouseListener, addMouseMotionListener, addMouseWheelListener, bounds, checkImage, checkImage, contains, createImage, createImage, createVolatileImage, createVolatileImage, dispatchEvent, enable, enableInputMethods, firePropertyChange, firePropertyChange, firePropertyChange, firePropertyChange, firePropertyChange, getBackground, getBounds, getColorModel, getComponentListeners, getComponentOrientation, getCursor, getDropTarget, getFocusCycleRootAncestor, getFocusListeners, getFocusTraversalKeysEnabled, getFont, getForeground, getGraphicsConfiguration, getHierarchyBoundsListeners, getHierarchyListeners, getIgnoreRepaint, getInputContext, getInputMethodListeners, getInputMethodRequests, getKeyListeners, getLocale, getLocation, getLocationOnScreen, getMouseListeners, getMouseMotionListeners, getMousePosition, getMouseWheelListeners, getName, getParent, getPeer, getPropertyChangeListeners, getPropertyChangeListeners, getSize, getToolkit, getTreeLock, gotFocus, handleEvent, hasFocus, hide, imageUpdate, inside, isBackgroundSet, isCursorSet, isDisplayable, isEnabled, isFocusable, isFocusOwner, isFocusTraversable, isFontSet, isForegroundSet, isLightweight, isMaximumSizeSet, isMinimumSizeSet, isPreferredSizeSet, isShowing, isValid, isVisible, keyDown, keyUp, list, list, list, location, lostFocus, mouseDown, mouseDrag, mouseEnter, mouseExit, mouseMove, mouseUp, move, nextFocus, paintAll, postEvent, prepareImage, prepareImage, remove, removeComponentListener, removeFocusListener, removeHierarchyBoundsListener, removeHierarchyListener, removeInputMethodListener, removeKeyListener, removeMouseListener, removeMouseMotionListener, removeMouseWheelListener, removePropertyChangeListener, removePropertyChangeListener, repaint, repaint, repaint, resize, resize, setBounds, setBounds, setComponentOrientation, setCursor, setDropTarget, setFocusable, setFocusTraversalKeysEnabled, setIgnoreRepaint, setLocale, setLocation, setLocation, setName, setSize, setSize, show, show, size, toString, transferFocus, transferFocusUpCycle`

**Methods inherited from class java.lang.Object**

`equals, getClass, hashCode, notify, notifyAll, wait, wait, wait`

**Constructor Detail**

### AttributeSelectionPanel

```
public AttributeSelectionPanel()
```

:   Creates the classifier panel


**Method Detail**

### setLog

```
public void setLog(Logger newLog)
```

:   Sets the Logger to receive informational messages

    :   **Specified by:**: `setLog` in interface `Explorer.LogHandler`
    :   **Parameters:**: `newLog` - the Logger that will now get info messages

---


### setInstances

```
public void setInstances(Instances inst)
```

:   Tells the panel to use a new set of instances.

    :   **Specified by:**: `setInstances` in interface `Explorer.ExplorerPanel`
    :   **Parameters:**: `inst` - a set of Instances

---


### capabilitiesFilterChanged

```
public void capabilitiesFilterChanged(Explorer.CapabilitiesFilterChangeEvent e)
```

:   method gets called in case of a change event

    :   **Specified by:**: `capabilitiesFilterChanged` in interface `Explorer.CapabilitiesFilterChangeListener`
    :   **Parameters:**: `e` - the associated change event

---


### setExplorer

```
public void setExplorer(Explorer parent)
```

:   Sets the Explorer to use as parent frame (used for sending notifications
    about changes in the data)

    :   **Specified by:**: `setExplorer` in interface `Explorer.ExplorerPanel`
    :   **Parameters:**: `parent` - the parent frame

---


### getExplorer

```
public Explorer getExplorer()
```

:   returns the parent Explorer frame

    :   **Specified by:**: `getExplorer` in interface `Explorer.ExplorerPanel`
    :   **Returns:**: the parent

---


### getTabTitle

```
public java.lang.String getTabTitle()
```

:   Returns the title for the tab in the Explorer

    :   **Specified by:**: `getTabTitle` in interface `Explorer.ExplorerPanel`
    :   **Returns:**: the title of this tab

---


### getTabTitleToolTip

```
public java.lang.String getTabTitleToolTip()
```

:   Returns the tooltip for the tab in the Explorer

    :   **Specified by:**: `getTabTitleToolTip` in interface `Explorer.ExplorerPanel`
    :   **Returns:**: the tooltip of this tab

---


### main

```
public static void main(java.lang.String[] args)
```

:   Tests out the attribute selection panel from the command line.

    :   **Parameters:**: `args` - may optionally contain the name of a dataset to load.


---


|  |  |  |
| --- | --- | --- |
| **Overview** \| **Package** \| **Class** \| **Tree** \| **Deprecated** \| **Index** \| **Help** \| **Weka's home** | |  |
| **PREV CLASS**   **NEXT CLASS** | **FRAMES**    **NO FRAMES**     **All Classes** |  |
| SUMMARY: NESTED \| FIELD \| CONSTR \| METHOD | DETAIL: FIELD \| CONSTR \| METHOD |  |


---
